# Who Falls After a Stroke? Evidence From a Prospective Stroke Cohort

**DOI:** 10.1111/ene.70678

**Published:** 2026-06-24

**Authors:** Anna Kufner, Yunyou Tang, Uchralt Temuulen, Ghadir Abbas, Torsten Rackoll, Ulrike Grittner, Daniel Kroneberg, Benedikt Weigl, Andrea A. Kühn, Martin Reich, Alexander H. Nave, Matthias Endres

**Affiliations:** ^1^ Charité – Universitätsmedizin Berlin, Corporate Member of Freie Universität Berlin and Humboldt‐Universität Zu Berlin Klinik Für Neurologie Mit Experimenteller Neurologie Berlin Germany; ^2^ Charité ‐ Universitätsmedizin Berlin, Corporate Member of Freie Universität Berlin and Humboldt‐Universität Zu Berlin Center for Stroke Research Berlin (CSB) Berlin Germany; ^3^ Department of Neurology University Hospital and Julius‐Maximilians‐University Würzburg Germany; ^4^ Berlin Institute of Health at Charité – Universitätsmedizin Berlin Berlin Germany; ^5^ QUEST Center for Responsible Research Berlin Institute of Health at Charité – Universitätsmedizin Berlin Berlin Germany; ^6^ Charité – Universitätsmedizin Berlin, Corporate Member of Freie Universität Berlin and Humboldt‐Universität Zu Berlin Institut für Biometrie und Klinische Epidemiologie Berlin Germany; ^7^ Partner Site Berlin German Center for Neurodegenerative Diseases (Deutsches Zentrum Für Neurodegenerative Erkrankungen, DZNE) Berlin Germany; ^8^ Partner Site Berlin German Center for Mental Health (Deutsches Zentrum Für Psychische Gesundheit, DZPG) Berlin Germany; ^9^ Partner Site Berlin German Centre for Cardiovascular Research (Deutsches Zentrum Für Herz‐Kreislauferkrankungen, DZHK) Berlin Germany

**Keywords:** falls, gait, lesion network mapping, mobility, stroke

## Abstract

**Background:**

Falls affect over 30% of stroke survivors within the first year, yet lesion‐related mobility and gait impairments underlying fall risk remain poorly understood. This study aimed to identify lesion‐derived functional networks associated with impaired mobility, gait, and fall risk in subacute stroke, and to determine whether disruption to these networks is associated with falls during the six‐month follow‐up.

**Methods:**

We analyzed data from 94 patients with disabling subacute ischemic stroke from the prospective Baptize cohort, an imaging sub‐cohort of the multicenter PHYS‐STROKE trial. Principal component (PC) analysis reduced seven mobility‐related and four gait‐related baseline variables into two composites: PC1‐Mobility and PC1‐Gait, explaining 56% and 82% of variance, respectively. PC1‐Mobility indexed global disability, whereas PC1‐Gait reflected spatiotemporal walking capacity. Lesion network mapping (LNM) identified functional networks associated with each domain. Patient‐reported falls up to six months post‐enrollment were the primary endpoint.

**Results:**

LNM revealed that the mobility‐related network predominantly involved cortical regions, whereas the gait‐related network was linked to subcortical and infratentorial connectivity. In binary multivariable logistic regression, network similarity scores were not associated with falls; only older age was significant (adjusted OR 1.08, 95% CI 1.02–1.16, *p* = 0.01). LNM of fall occurrence identified a cortical network with significant spatial overlap with the mobility‐related network (*p* < 0.001).

**Conclusion:**

This exploratory, hypothesis‐generating study identified distinct functional networks for post‐stroke mobility and gait impairment. Falls may be more closely linked to disruptions in cortical networks supporting voluntary motor control and whole‐body coordination than to subcortical gait‐modulating structures, potentially informing fall risk stratification and targeted prevention.

**Trial Registration:**

ClinicalTrials.gov identifiers: BAPTISe: NCT01954797, PHYS‐STROKE: NCT01363856

## Graphical abstract

Using principal component analysis and lesion network mapping, we identified lesion‐derived functional networks underlying mobility impairment, gait dysfunction, and fall risk in a subacute stroke cohort, and characterized the network disruptions associated with fall risk. Fall risk was more strongly associated with cortical network disruptions involving voluntary motor control and whole‐body coordination than with subcortical gait‐modulating network patterns.

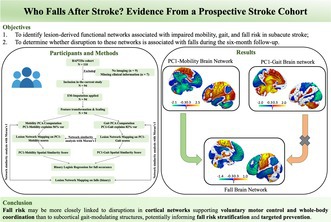

## 1 Introduction

Over one‐third of stroke survivors experience at least one fall within the first year after stroke, with approximately one‐third of these falls requiring medical intervention [1]. Falls in this patient population are associated with prolonged hospitalization, increased morbidity and mortality, and a significant decline in post‐stroke quality of life [1, 2]. Despite their clinical relevance, however, falls in stroke survivors specifically remain understudied with no evidence of effective therapy for secondary prevention of falls after stroke [3, 4].

Previous observational studies have identified several risk factors for falls in stroke populations, including advanced age, sedative medication, neuropsychiatric symptoms such as depression and cognitive impairment, and the degree of functional disability [1, 5, 6]. Reported risk factors in stroke survivors largely overlap with those observed in an age‐matched general population [3]. However, certain stroke‐specific factors—such as unilateral weakness, impaired coordination, and gait dysfunction—are likely to play a disproportionately larger role in post‐stroke falls [7].

Ischemic stroke can disrupt critical motor pathways, leading to both general mobility impairment and more nuanced gait dysfunction in up to 50% of stroke survivors [8]. Despite the clinical relevance of post‐stroke falls, predicting individual fall risk remains challenging. Previous lesion studies suggest that damage to the corticospinal tract and adjacent structures in the capsular‐putaminal region plays a role in supraspinal gait control [9, 10]. Similarly, a recent lesion‐network mapping study identified a so‐called ‘gait network’, in which lesions functionally connected to the anterior cingulate cortex, midbrain, and pons were associated with gait impairment [11]. Given the involvement of multiple systems beyond the corticospinal tract, and substantial interindividual differences in compensatory capacity, lesion location alone is likely insufficient to fully explain or predict fall risk [10, 11, 12]. Collectively, our understanding of the neuroanatomical basis and complexity of post‐stroke motor and gait dysfunction remains limited—particularly regarding their specific contribution to fall risk. A better understanding of stroke‐specific risk factors for falls could improve identification of high‐risk fallers and lead to targeted prevention strategies and individualized rehabilitation planning.

Therefore, we here analyzed a well‐characterized prospective cohort of patients with disabling ischemic stroke who underwent mobility and gait performance assessments at study enrollment and were monitored for fall occurrence over a six‐month period. The mobility domain captured global disability and basic functional mobility in daily life, whereas the gait domain represented spatiotemporal aspects of walking capacity and efficiency. Our objectives were to: (1) apply lesion‐network mapping to identify networks associated with impaired mobility, gait, and falls; and (2) determine whether lesion‐induced disruption to these networks is associated with the risk of falling after stroke.

## 2 Materials and Methods

### 2.1 Participants

This study is an exploratory, secondary analysis of data from the prospective observational cohort Baptize (Biomarkers and Perfusion‐Training‐Induced Changes After Stroke; ClinicalTrials.gov identifier: NCT01954797 [13]) embedded in the multicenter, randomized controlled trial PHYS‐STROKE (Physical Fitness Training in Patients with Subacute Stroke; ClinicalTrials.gov NCT01953549 [14]). Patients were enrolled if they had experienced a subacute ischemic stroke (5–45 days after symptom onset) and had a Barthel Index score ≤ 65 at screening, with the primary aim of evaluating the effects of aerobic exercise on mobility and activities of daily living during the subacute post‐stroke phase. Patients underwent clinical assessments at study enrollment (baseline), directly after the 4‐week intervention period, and at six‐month follow‐up. For inclusion in the present analysis, participants were required to have available pre‐intervention magnetic resonance imaging (MRI). All participants provided written informed consent. The study was conducted in accordance with the Declaration of Helsinki and approved by the local ethics committee in Berlin (EA1/137/13).

### 2.2 Imaging

All patients received a standardized stroke imaging protocol with a 3 T MRI scanner, as detailed in the original study protocol [13]. Lesions were manually delineated on fluid‐attenuated inversion recovery (FLAIR) images by a neurologist using the semi‐automated lesion masking toolbox Clusterize [15]. All lesion masks were reviewed by an expert radiologist. The resulting lesion masks were co‐registered to brain‐extracted and bias‐corrected b0 images using the Brain Extraction Tool (BET) from the FMRIB Software Library (FSL) [16]. Lesion masks were then normalized to MNI152 standard space (1 × 1 × 1 mm resolution) [17] using the Symmetric Normalization (SyN) algorithm implemented in the Advanced Normalization Tools (ANTs) for Python [18].

### 2.3 Clinical Assessment

Our primary clinical endpoint was the occurrence of patient‐reported falls during the six‐month period following study enrollment. Falls were assessed during scheduled follow‐up visits, where treating physicians asked participants whether they had experienced a fall since the last contact. While falls could occur during the 4‐week intervention period, no falls were reported during supervised intervention sessions. Outside of these visits, fall ascertainment was not systematically recorded. Fall occurrence was recorded as a binary variable. To investigate potential neural correlates of fall risk, we focused on two key baseline domains: Mobility and gait, which served as behavioral inputs for subsequent imaging analyses.

Mobility was assessed using a composite of seven clinical variables that capture overall functional dependency status and general mobility. All the following measures were administered by trained study assessors (physiotherapists and occupational therapists) who collected data from chart reviews at baseline visits and carried out standardized outcome assessments [14]. These included the Modified Rankin Scale (mRS) [19], a clinician‐rated scale ranging from 0 (no symptoms) to 6 (death) in which functional dependence is assessed; the EQ‐5D‐5L mobility subdomain [20], a patient‐reported scale from 1 (no problems) to 5 (unable to walk); and the Rivermead Mobility Index (RMI) [21], which evaluates the ability to perform functional tasks such as transfers, walking, and stair climbing, scored 0–15 with higher scores indicating better mobility. In addition, we recorded scores on three mobility‐related items from the Barthel Index (BI), walking, stair climbing, and transfers, with higher scores indicating greater independence [22]. Finally, the use of a walking aid (yes/no) during the walking test was documented [14].

Gait was assessed using four performance‐based parameters that reflect both short‐distance and endurance walking ability. The 10‐Meter Walk Test (10MWT) [23] required participants to walk 14 m at maximum speed; only the middle 10 m were timed, with 2 m for acceleration and deceleration at each end. Time was recorded using an electronic stopwatch and verified with a light‐beam trigger device, and two trials were performed with the mean used to calculate gait speed (m/s); a higher speed indicates better performance. The number of steps and duration were additionally recorded. The Six‐Minute Walk Test (6MWT) [23] recorded the total distance walked in 6 min as a measure of submaximal aerobic capacity and walking endurance, with greater distance indicating better performance. After the variable transformations described in the following Data Preprocessing and Dimensionality Reduction section, higher values of the above‐mentioned mobility and gait variables reflected worse function, with zero corresponding to clinically normal performance.

### 2.4 Data Preprocessing and Dimensionality Reduction

**FIGURE 1:**
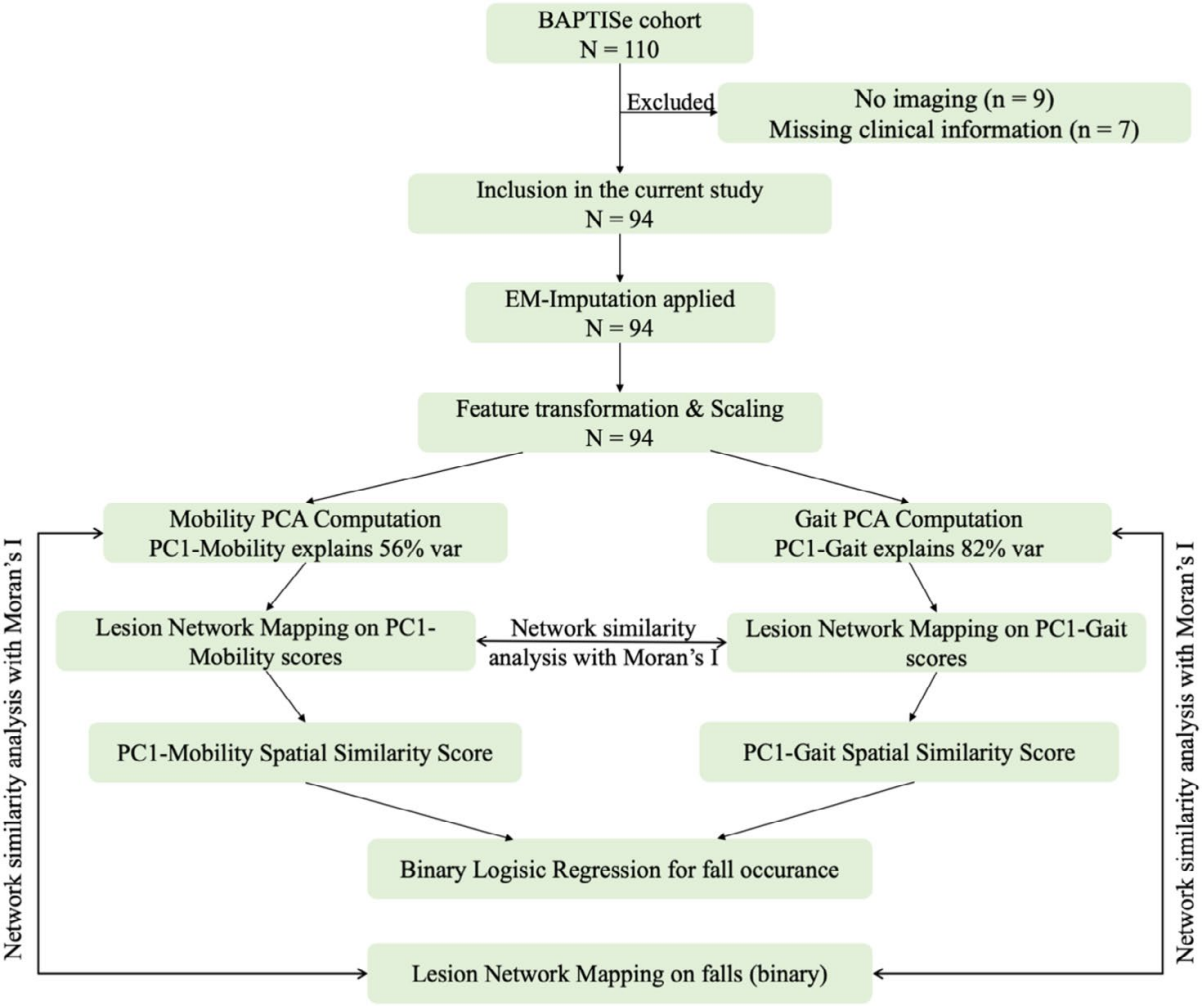
Flow‐chart of the data analysis pipeline.

For a flow‐chart depicting our analysis pipeline from data pre‐processing to imaging analyses, refer to Figure 1. All preprocessing and dimensionality‐reduction steps with principal component analysis (PCA) were performed separately for the mobility domain (seven variables: mRS, RMI, EQ‐5D‐5L mobility sub‐domain, and four Barthel sub‐scores) and the gait domain (four variables: 10‐m walk speed, number of steps, walking time, and 6‐min walk distance).

To harmonize directionality of the clinical variables, each variable was transformed so that higher values reflected worse function and zero corresponded to clinically normal performance. Accepted clinical reference values (e.g., 300 m for the 6‐min walk, 1.2 m s^−1^ for gait speed [24, 25]) or full‐scale maxima for ordinal measures were subtracted; negative residuals—representing better‐than‐normal performance—were truncated to zero to avoid artificial inverse correlations. For a detailed list of how all mobility and gait variables were transformed, refer to Table S1. All variables were then z‐normalized. Before dimensionality reduction, Pearson correlation matrices were computed to assess intercorrelations among variables within each domain (Figure S1). The observed collinearity—moderate to high within the mobility domain (*r* = 0.25–0.71) and high within the gait domain (r = 0.66–0.95)—supported the use of PCA, which accommodates correlated inputs and transforms them into orthogonal components, thereby reducing redundancy while retaining the major sources of variance. Missingness across the 11 gait‐ and mobility‐related variables ranged from 0% to 14.9% (median≈1%); the 6‐Minute Walk Test distance had the most missing entries (*n* = 14). Missing entries were imputed via median‐initialized expectation–maximization (EM; 50 iterations, tol = 1 × 10^−4^) implemented in Python 3.11 (scikit‐learn 1.4), an approach shown to preserve principal‐component structure under moderate missingness [26]. Complete‐case sensitivity analyses reproduced the same loading pattern, confirming that the imputation did not bias the PCA solution. Sampling adequacy was confirmed (Kaiser–Meyer–Olkin = 0.89) and Bartlett's test indicated sufficient shared variance (χ^2^ = 783.3, *p* < 0.0001). Component retention followed Horn's parallel analysis [27]: 5000 Monte‐Carlo permutations were generated, and components whose observed eigenvalues exceeded the 95ᵗʰ‐percentile of the random distribution were retained. Scree‐plot inspection (“elbow”) and Kaiser's λ > 1 heuristic served as secondary, non‐binding checks. No rotation was applied, preserving PC1 orthogonality for subsequent lesion‐network mapping.

### 2.5 Lesion‐Network Mapping (LNM) of Gait, Mobility, and Falls

LNM was conducted using the Lead Mapper from Lead‐DBS (lead‐dbs.org) [28], as previously described. Briefly, for each patient, a functional connectivity profile was generated from their lesion mask using a normative resting‐state fMRI connectome derived from 1000 healthy individuals [29, 30]. The resulting maps contain Pearson correlation coefficients, which were subsequently Fisher z‐transformed.

First, separate LNM analyses were performed for the two behavioral clusters of interest: Gait and mobility. For each, the PC1 from the respective domain was used as the independent variable. Statistical testing was performed using FSL's randomize tool with 5000 permutations and two contrasts: +1 for regions where connectivity was associated with impairment, and −1 for regions associated with spared function. Multiple comparisons were corrected using family‐wise error (FWE) correction and threshold‐free cluster enhancement (TFCE). Resulting T‐score maps were overlaid on structural standard atlases to identify connected anatomical regions implicated in gait and mobility dysfunction, as described in detail previously [31, 32].

According to the description in a previous study [32], spatial similarity scores for gait and mobility were derived from the uncorrected T‐value LNM maps based on the individual connectivity fingerprints of each patient. Specifically, the voxelwise Pearson correlation coefficient between each patient's lesion connectivity map and the symptom‐specific network map was computed, and then Fisher‐z transformed. This quantified how strongly the spatial pattern of a patient's lesion‐related connectivity aligned with the respective symptom network. Higher spatial similarity scores, therefore, indicate a greater degree of overlap between an individual's lesion connectivity pattern and the network associated with gait or mobility outcomes. Finally, to identify independent predictors of fall risk, we performed separate binary logistic regression analyses including age, treatment group (physical fitness vs. relaxation [14]), and either the mobility‐related or gait‐related spatial similarity score.

Lastly, we performed LNM for our primary outcome of interest: Falls. Again, in this study, falls refer to events reported during the intervention and follow‐up period; importantly, no falls occurred during supervised intervention sessions. Fall ascertainment outside scheduled study visits was not systematic and relied on patient report at follow‐up assessments. Fall occurrence was modeled as a binary dependent variable in FSL's randomize tool, applying 5000 permutations and two contrasts: [1, −1] to identify regions functionally connected to lesions associated with the occurrence of falls, and [−1, 1] to identify regions connected to lesions in patients without falling. This analysis was adjusted for age, treatment group (based on the results of the prior binary logistic regression analysis), and baseline lesion volume. To evaluate the spatial similarity and dissimilarity between the resulting fall network and the previously derived PC1‐mobility and PC1‐gait networks, we used voxel‐wise Spearman rank correlation. Statistical testing of these spatial associations was determined via Moran's spectral randomization (MSR) as implemented in the BrainSpace toolbox [33, 34]. MSR exploits the eigenvectors of a spatial weight matrix to compare the observed correlation against a null distribution of spatially autocorrelated surrogate maps. We defined the spatial weights as the inverse squared inter‐voxel distance to avoid arbitrary distance thresholds and to prevent over‐weighting distant voxels. For each comparison, 10,000 surrogate maps were generated, and *p*‐values were computed as the proportion of null correlations greater than or less than the observed correlation [35, 36, 37]. All networks were down‐sampled to 4‐mm isotropic resolution for this analysis only, to improve computational efficiency while preserving spatial structure.

### 2.6 The Use of Artificial Intelligence Generated Content (AIGC) Tools

We acknowledge the use of OpenAI's ChatGPT‐4 for assisting in writing and correcting the code used in this study. The tool was used to enhance code efficiency and troubleshoot errors during the analysis.

## 3 Results

### 3.1 Patient Cohort Description

**FIGURE 2:**
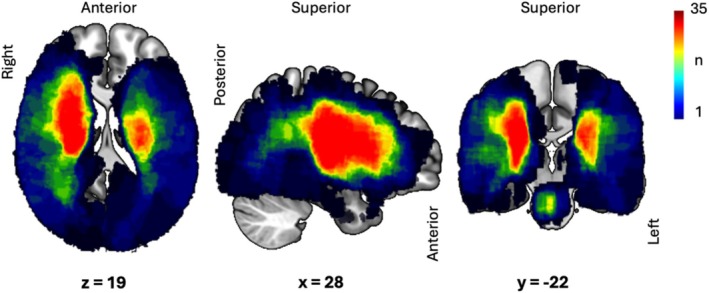
Heat‐map showing lesion overlap from all patients included in the final analysis (*N* = 94) overlaid on a 100 μm brain scan in MNI space. The color bar reflects the number of ischemic brain lesions overlapping at the voxel level, from blue to red, showing a maximal overlap of 35 lesions, and depicts the wide distribution of lesions across 94 patients.

A total of 110 patients were enrolled in the Baptize study [38], of which 94 met our inclusion criteria. The mean age was 69 years (standard deviation [SD]: 11 years), and the median NIHSS score at admission was 9 (interquartile range [IQR]: 6–12). The median of post‐stroke inclusion time window was 25.5 (interquartile range [IQR]: 14–33). A lesion heatmap illustrating the distribution of all ischemic brain lesions included in the analysis is shown in Figure 2. During the six‐month follow‐up period, 17 patients (18%) reported a fall. Fallers were older than non‐fallers (mean age in years 75 vs. 67; *p* < 0.05), but the post‐stroke inclusion window did not differ significantly between fallers and non‐fallers. Fallers had statistically significantly higher ARWMC scores compared to non‐fallers (median 8, IQR 6–14 vs. 5, IQR 4–8; *p* < 0.05). Lesion volume did not substantially differ between fallers and non‐fallers. A detailed summary of patient demographics, including cerebrovascular risk factors, stroke etiology, white matter hyperintensities, and lesion volume, is provided in Table S2. A description of the characteristics of fallers (*n* = 17) is provided in Table S3.

### 3.2 Dimensionality Reduction of Mobility and Gait via PCA

In the mobility domain, PC1 accounted for 56% of the total variance; in the gait domain, PC1 explained 82.1%. All variables loaded positively on their respective PC1 (range: 0.31–0.53 for mobility, 0.47–0.53 for gait), indicating that higher scores consistently reflected greater functional impairment (Figure S2). Horn's parallel analysis with 5000 iterations (Figure S3) confirmed that only PC1 exceeded the 95th percentile of the null distribution in both the mobility (eigenvalue = 3.96) and gait (eigenvalue = 3.32) domains, supporting the retention of a single component per domain for subsequent analyses. Visual inspection of scree plots revealed a clear elbow after the first component, and Kaiser's criterion likewise supported the one‐component solution.

### 3.3 Mobility and Gait Networks From LNM and Fall Risk

**FIGURE 3:**
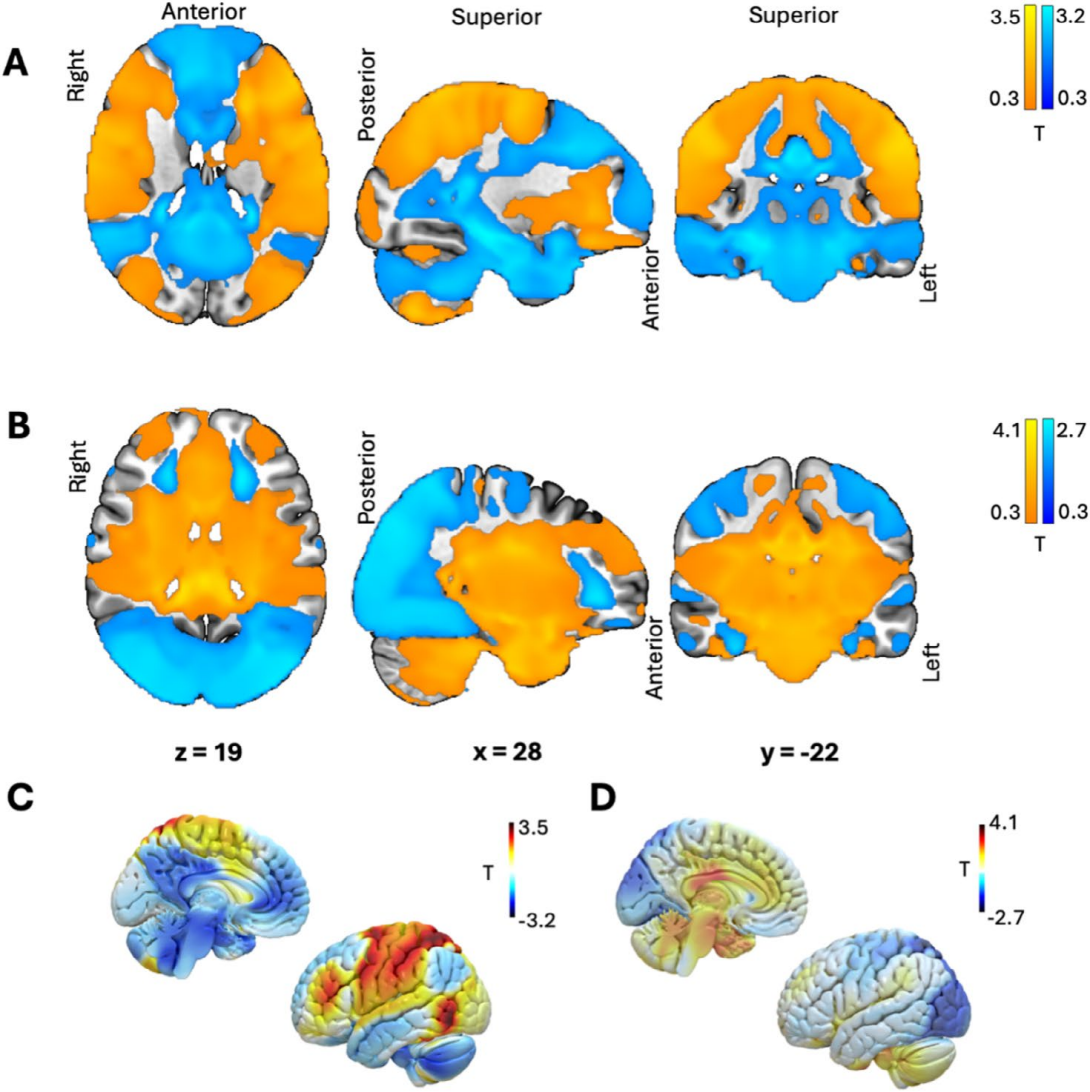
Lesion‐network maps for PC1‐Mobility and PC1‐Gait (N = 94). (A) Uncorrected T‐score map showing regions functionally connected to ischemic brain lesion locations associated with mobility impairment, using PC1 scores from the mobility domain as the continuous variable; 1‐pFWE 0–0.82. Of note, these maps represent uncorrected T‐score distributions and are presented for exploratory visualization purposes only; they do not reflect findings that survived FWE correction. (B) Uncorrected T‐score map for gait impairment, based on PC1 scores from the gait domain (1‐pFWE 0–0.98). Color bars indicate T‐values, with red–yellow representing positive T values and blue–black representing negative T values. (C) Surface‐rendered map of the mobility network (PC1‐Mobility). (D) Surface‐rendered map of the gait network (PC1‐Gait). All maps are overlaid on the MNI152 100 μm brain template. Color bars indicate T‐values, with values increasing from dark blue to dark red.

Uncorrected T‐score lesion network maps for both behavioral domains, mobility and gait, are shown in Figure 3. The PC1‐Mobility network comprises predominantly cortical regions, including key sensorimotor areas such as the pre‐ and postcentral gyri, as well as associative and integrative regions like the superior and middle frontal gyri, insular cortex, and supramarginal gyrus; 1‐pFWE values ranged from 0 to 0.82. Notably, the PC1‐Mobility network did not involve subcortical or cerebellar structures. The PC1‐Gait network showed widespread involvement of subcortical regions—including the bilateral thalamus—as well as frontal cortical regions, including the precentral gyrus, paracingulate and cingulate cortices, and insular cortex; 1‐pFWE values ranged from 0 to 0.98. The network also included connectivity to infratentorial regions, including the brainstem and left cerebellar regions (left Crus I and II). For a full list of involved regions within the PC1‐Mobility and PC1‐Gait associated network, refer to Table 1.

**TABLE 1:** Anatomical regions functionally connected to uncorrected t‐score maps of PC1‐Mobility and PC1‐Gait. this table lists cortical, subcortical, and cerebellar regions identified within the functional symptom networks for PC1‐Mobility and PC1‐Gait. regions were extracted using the Harvard‐Oxford cortical (HOOC) and subcortical (HOOS) structural atlases, as well as the FNIRT‐normalized cerebellar atlas, all in MNI152 standard space.

	HOOC	HOOS	Atlas of the human cerebellum
**PC1‐Mobility Network**	Frontal Pole	—	—
	Insular Cortex		
	Superior Frontal Gyrus		
	Middle Frontal Gyrus		
	Precentral Gyrus		
	Middle Temporal Gyrus, temporooccipital part		
	Inferior Temporal Gyrus, temporooccipital part		
	Postcentral Gyrus		
	Superior Parietal Lobule		
	Supramarginal Gyrus, anterior division		
	Supramarginal Gyrus, posterior division		
	Lateral Occipital Cortex, superior division		
	Lateral Occipital Cortex, inferior division		
	Juxtapositional Lobule Cortex (formerly Supplementary Motor Cortex)		
	Central Opercular Cortex		
	Occipital Pole		
**PC1‐Gait Network**	Frontal Pole	Left Thalamus	Left Crus I
	Insular Cortex	Brain‐Stem	Left Crus II
	Superior Frontal Gyrus	Right Thalamus	
	Precentral Gyrus		
	Temporal Pole		
	Paracingulate Gyrus		
	Cingulate Gyrus, anterior division		
	Cingulate Gyrus, posterior division		
	Frontal Orbital Cortex		
	Central Opercular Cortex		

In univariable and multivariable binary logistic regression analysis for fall occurrence, older age (adjusted odds ratio [OR] 1.08, 95% Confidence Interval [CI] 1.02–1.15; *p* = 0.01) was independently associated with a higher risk of falls in both models when PC1‐Mobility and PC1‐Gait spatial similarity scores were included separately. Treatment group (i.e., intervention‐arm physical activity vs. relaxation) was also associated with a higher risk of falls, as reported in the original trial results (adjusted OR 3.1, 95% CI 0.92–10.31, *p* = 0.068), although this was not statistically significant. Importantly, patient‐specific connectivity to the mobility and gait network was not associated with higher fall risk in either univariable or multivariable regression analyses (Table S4). The multivariable model including the PC1‐Mobility spatial similarity score showed slightly better overall fit (pseudo *R* ^2^ = 0.145, AIC = 83.67) compared with the model including the PC1‐Gait spatial similarity score (pseudo *R* ^2^ = 0.139, AIC = 84.14), although the difference was negligible.

**FIGURE 4:**
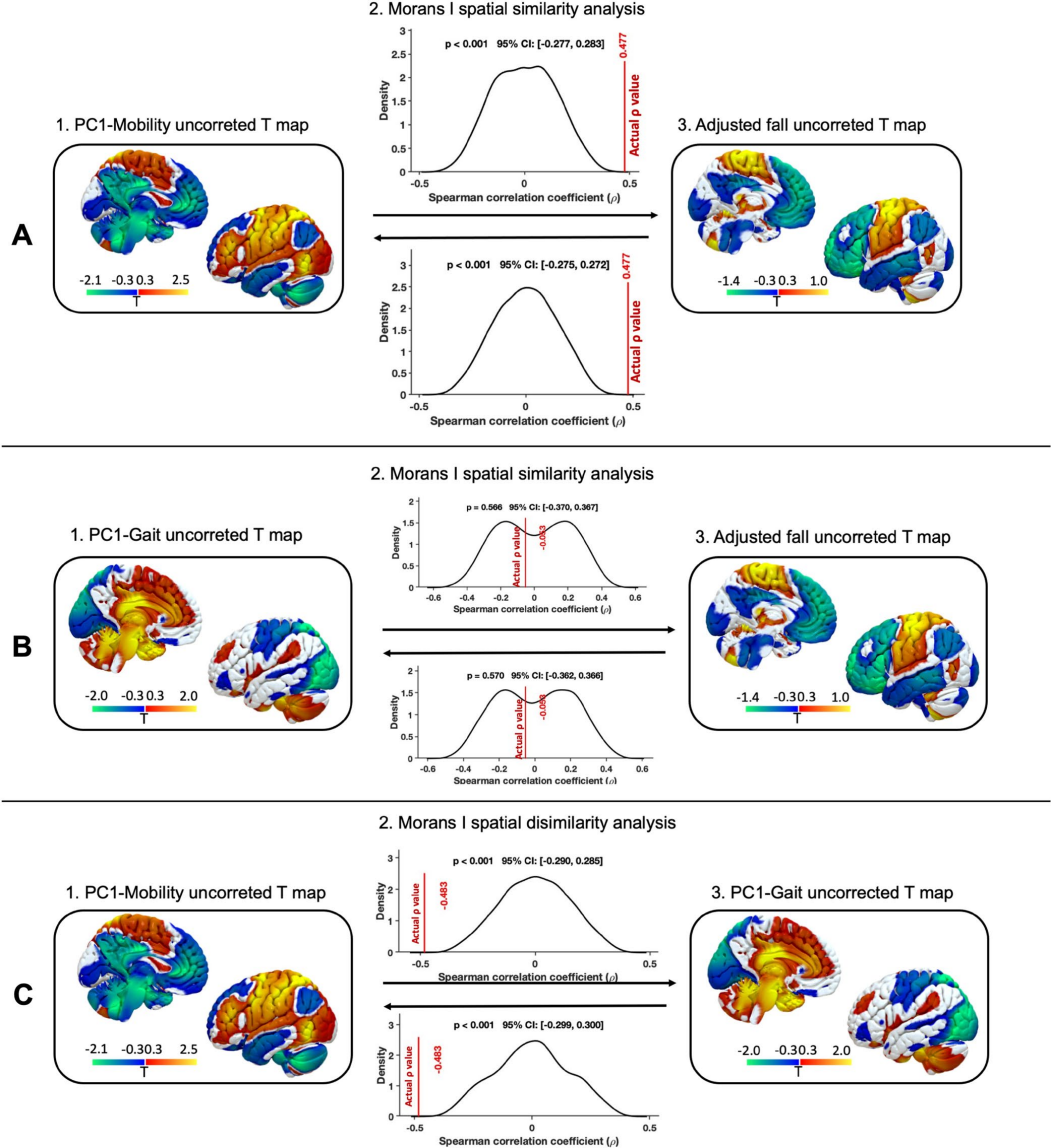
Similarity and dissimilarity between PC1‐Mobility and PC1‐Gait brain networks (*N* = 94), and adjusted fall network (*N* = 89). Whole‐brain network similarity analysis using Moran's I and 10,000 spatial surrogates to compare the spatial similarity or dissimilarity between the network associated with (A) PC1‐Mobility and the network associated with fall, adjusted for age, lesion volume, and treatment group; (B) PC1‐Gait and the network associated with fall, adjusted for age, lesion volume, and treatment group; (C) PC1‐Mobility (*N* = 94) and PC1‐Gait (*N* = 94). The map at the arrow origin is permuted, compared to the unpermuted target map. Network maps are statistically significantly more alike than expected by chance, indicating a high similarity. All maps are overlaid on the MNI152 100 μm brain template. Color bars indicate uncorrected T‐values, with red–yellow representing positive connectivity and blue–green representing negative connectivity.

Although the PC1‐mobility and PC1‐gait network were not independently associated with fall risk in the regression analysis described above, we ran an additional, exploratory LNM analysis focused on the primary outcome of falls (*N* = 89)—adjusted for age, lesion volume, and treatment group. Here, fall occurrence after stroke was associated with lesions functionally connected to a bilateral network encompassing primary and secondary sensorimotor regions (pre‐ and postcentral gyri), parietal areas (superior parietal lobule and supramarginal gyrus), insular cortices, and the left cerebellar lobules; however, no voxels survived FWE correction. Full atlas query results from the fall network are provided in Table S5. To assess whether the PC1‐Mobility and PC1‐Gait networks were spatially related to each other and to the fall‐associated brain network, we used Moran's I with 10,000 spatial surrogates to quantify spatial similarity. The results demonstrated that the overlap between the uncorrected PC1‐Mobility T‐score map and the fall‐associated network was greater than expected by chance (Spearman *r* = 0.48, *p* < 0.001), as shown in Figure 4A. In contrast, the spatial similarity between the PC1‐Gait and fall‐associated uncorrected T‐score maps was not statistically significant (Figure 4B), with *p* > 0.05. Furthermore, the PC1‐Gait and PC1‐Mobility uncorrected T‐score maps were statistically dissimilar (Spearman *r* = −0.48, *p* < 0.001), as shown in Figure 4C.

## 4 Discussion

In this prospective cohort of patients with disabling ischemic stroke, we applied LNM to investigate the functional brain networks underlying mobility, gait, and falls, and their relationship to post‐stroke fall risk. Impaired mobility was associated with lesions functionally connected to predominantly cortical regions, including primary and secondary sensorimotor areas, suggesting disruption of cortical motor execution and integration networks. In contrast, gait dysfunction was linked to lesions connected with subcortical and infratentorial structures—most notably the thalamus, brainstem, and cerebellum—highlighting the importance of subcortical circuits and cerebellar modulation in the coordination and automation of gait. The network associated with mobility impairment was significantly spatially similar to the network associated with fall risk, indicating that lesion‐induced disruptions within the mobility‐related network may contribute to increased fall susceptibility.

This study population consisted of patients with disabling ischemic stroke with moderate to high stroke severity (median NIHSS score of 9) and relatively large ischemic brain lesion volumes (median 11 mL). Baseline clinical and demographic characteristics of the analyzed cohort (*N* = 94) were consistent with those reported in the main PHYS‐STROKE trial [14]. Despite this, the overall fall occurrence was relatively low (18% within six months), compared to previous reports ranging from 30% to 50% up to one year [1, 2, 5]. This discrepancy is likely due to the short observation period, but may also be attributed to the structured inpatient rehabilitation programs of patients within the Baptize cohort, which likely reduced fall risk through close clinical monitoring.

Notably, 11 of the 17 fallers were either wheelchair‐dependent or not mobile at all, compared to 57 of 77 non‐fallers (Table S2). Similarly, only one faller was able to transfer independently between bed and chair, vs. 11 non‐fallers. These findings suggest that limited mobility alone does not necessarily protect against falls. Interestingly, fallers also showed higher median spasticity scores (REPAS score: 12 [IQR 2–14]) compared to non‐fallers (6 [IQR 2–11]), though this difference was not statistically robust (*p* = 0.396). This may indicate a trend toward greater motor impairment in fallers, but further studies are needed to clarify this association.

Fallers showed a higher white matter lesion load, as measured by the ARWMC score on baseline MRI (Table S2). This association may be partially explained by the co‐linear relationship between advanced age and increased white matter burden [39, 40]. However, white matter lesions also reflect underlying small vessel disease and may disrupt critical motor and sensorimotor pathways—such as the corticospinal tract and thalamocortical loops—potentially contributing to impaired mobility and balance control after stroke [41, 42]. Although the impact of white matter lesions on fall risk was not a primary aim of the current study, this association warrants further investigation in future studies.

While lesions to the corticospinal tract often result in hemiparesis or hemiplegia with characteristic hemiplegic gait patterns, damage to subcortical structures involved in motor modulation rather than direct motor execution may preferentially impair gait control while leaving general mobility relatively intact [8, 43, 44]. In line with prior work, mobility and gait were treated as related but distinct constructs in this study. Mobility refers to the ability to move freely and efficiently in everyday functional tasks such as transfers and short‐distance ambulation, whereas gait encompasses walking speed, endurance, and coordination assessed under standardized conditions [45]. This distinction likely underlies the distinct symptom networks (significantly dissimilar spatial pattern in Moron's I analysis: Spearman *r* = −0.48, *p* < 0.001, Figure 4C) observed for the composite mobility score (PC1‐Mobility) and the gait score (PC1‐Gait; Figure 3).

In a binary logistic regression analysis for falls up to six months post‐stroke, only advanced age was independently associated with increased falls (adjusted OR 1.08, 95% CI 1.02–1.15), which stands in line with previous studies [3, 46]. As reported in the primary trial results, patients allocated to the physical fitness intervention group had a higher risk of falling compared to those in the relaxation group (adjusted OR 3.98, 95% CI 1.09–14.57 in PC1‐Mobility model and adjusted OR 3.67, 95% CI 1.09–14.57 in PC1‐Gait model). Similar rehabilitation trials in small stroke cohorts have also noted an increased frequency of adverse events, including falls, in intervention arms—highlighting the need for further investigation into the safety of early post‐stroke mobilization strategies [47, 48]. While we hypothesized that individual connectivity fingerprints to mobility and gait networks would increase fall risk, patient‐specific spatial similarity scores were not independently associated with falls in this analysis (Table S4). Given that falls were assessed during a defined rehabilitation phase in the current study and relied on patient self‐report, caution is warranted in generalizing these findings to long‐term post‐stroke fall risk.

An additional network mapping analysis of fall occurrence identified a predominantly bilateral cortical network involving primary and secondary sensorimotor, parietal, opercular, insular, and occipital regions, with additional contributions from the right thalamus and left cerebellar lobule VI. Whole‐brain spatial similarity analysis demonstrated a statistically significant overlap between this fall‐associated network and the PC1‐Mobility network (Figure 4). These findings are compatible with the hypothesis that post‐stroke falls may be more closely linked to disruptions in cortical networks supporting voluntary motor control and sensorimotor integration than to subcortical systems primarily involved in automated gait modulation [43].

This network‐level finding aligns with clinical and experimental evidence indicating that post‐stroke imbalance frequently arises from impaired multisensory integration, particularly of visual and somatosensory inputs [49]. Individuals with hemiplegia often rely more heavily on visual feedback to maintain postural stability, reflecting compensatory strategies that may be insufficient under complex or dynamic conditions. In parallel, hemiparesis‐related gait abnormalities—such as reduced paretic propulsion, impaired single‐limb support, and spatiotemporal asymmetries—necessitate energetically inefficient compensations that further increase fall susceptibility [12]. Emerging concepts of the somato‐cognitive organization of the motor cortex provide an additional framework through which cortical lesions may disrupt higher‐order motor planning and coordination, thereby contributing to fall risk beyond simple deficits in locomotor execution [43].

Together, these observations support the notion that post‐stroke falls are driven not solely by gait impairment, but by broader disturbances in cortical sensorimotor networks underlying balance, coordination, and whole‐body control. This distinction may facilitate more refined fall‐risk stratification based on individual lesion characteristics. For example, patients with lesions affecting networks critical for mobility may benefit from more intensive, targeted physiotherapy focusing on fall prevention and safe ambulation. In addition, the identified networks may represent potential targets for neuromodulatory interventions (non‐invasive or invasive [50]), although these findings require validation in independent cohorts.

### 4.1 Strengths and Limitations

First and foremost, it is important to note that this is an exploratory analysis and was not pre‐defined in the statistical analysis plan of the Baptize study. Furthermore, information on falls was limited to a defined rehabilitation interventional phase; data on falls were binary (yes/no), without details on number, severity, or consequences (e.g., hospitalization). Additionally, the precise timing of each fall was not recorded, as falls were assessed only at follow‐up visits via retrospective self‐report.

A further limitation relates to the assessment of mobility and gait using standard clinical scales rather than more specialized or instrumented measures (e.g., Functional Gait Assessment or quantitative kinematics). While the 10‐Meter Walk Test and 6‐Minute Walk Test are well‐validated and widely recommended in stroke research [23, 51], they primarily capture global aspects of walking performance and may not fully reflect qualitative gait abnormalities. In addition, the mobility domain comprised a heterogeneous set of measures, reflecting different aspects of functional independence, which may introduce variability in how mobility impairment is represented. Furthermore, although mobility and gait were operationalized as distinct domains and supported by both statistical (PCA) and neuroanatomical (LNM) dissociation, some degree of conceptual and measurement overlap between these constructs cannot be excluded.

Another limitation relates to the wide inclusion window of 5 to 45 days post‐stroke from the original PHYS‐STROKE trial design [14]. This variability may have introduced heterogeneity in baseline clinical status, as patients enrolled earlier may have greater potential for spontaneous neurological recovery compared with those enrolled later [52]. Consequently, baseline mobility and gait assessments—which served as inputs for the principal component analysis—may not reflect equivalent functional states across patients, potentially affecting the distribution of PC1 scores. Furthermore, lesion characterization may have varied with imaging timing, as infarct evolution can continue over the first weeks post‐stroke, potentially introducing variability into LNM analyses [53]. Nonetheless, the post‐stroke inclusion window did not differ significantly between fallers and non‐fallers in our cohort, minimizing the potential impact of this variability. Similarly, the PHYS‐STROKE investigators reported that subgroup analyses comparing early vs. late enrolment detected no significant differences in treatment effects [14]; however, residual confounding by time since stroke onset cannot be fully excluded from our secondary LNM analysis.

Additionally, an inherent methodological limitation of LNM is the reliance on normative connectomes derived from healthy young adults, which may not fully capture functional connectivity patterns in older, neurologically impaired populations such as stroke survivors.

However, the study also offers notable strengths. It draws on a well‐characterized cohort from a randomized controlled trial, with detailed and standardized assessments of gait performance and mobility. The inclusion of moderately to severely affected patients increases the likelihood of detecting meaningful lesion effects. Unlike prior LNM studies that rely on a single behavioral covariate, this analysis used PCA to reduce dimensionality across multiple clinical variables while preserving relevant clinical information. To our knowledge, this is the first study to investigate the prognostic value of data‐driven gait and mobility networks in ischemic stroke patients concerning future fall risk.

## 5 Conclusion

Stroke‐specific contributors to fall risk remain poorly understood. In this exploratory study, we identified lesion‐derived functional networks whose disruption may be more strongly associated with post‐stroke falls—particularly networks involved in voluntary motor execution, action integration, and whole‐body coordination—than those affecting subcortical regions modulating automated gait. These findings are hypothesis‐generating and require validation in larger, independent cohorts. If confirmed, they may support lesion‐informed risk stratification and guide more targeted fall prevention strategies during the early recovery phase.

## Author Contributions

**Torsten Rackoll:** data curation, writing – review and editing. **Ghadir Abbas:** writing – review and editing. **Yunyou Tang:** conceptualization, methodology, formal analysis, writing – original draft, writing – review and editing. **Anna Kufner:** conceptualization, methodology, writing – original draft, writing – review and editing, supervision. **Matthias Endres:** conceptualization, supervision, writing – review and editing. **Martin Reich:** writing – review and editing. **Andrea A. Kühn:** writing – review and editing. **Ulrike Grittner:** writing – review and editing. **Alexander H. Nave:** data curation. **Uchralt Temuulen:** formal analysis, methodology, writing – review and editing. **Daniel Kroneberg:** writing – review and editing. **Benedikt Weigl:** writing – review and editing.

## Funding

This work was supported by the Berlin Institute of Health; the NeuroCure Exzellenzcluster; Gemeinnützige Hertie‐Stiftung (Hertie Foundation); the Deutsche Forschungsgemeinschaft (EXC‐2049‐390688087); The Collaborative Research Center ReTune Transregional Collaborative Research Centre (295‐424778381, B07 Project); the Bundesministerium für Bildung und Forschung; the Corona‐Stiftung; the Else Kröner‐Fresenius‐Stiftung; and the Deutsches Zentrum für Herz‐Kreislauferkrankungen, DZHK.

## Conflicts of Interest

The author(s) declare the following potential conflicts of interest concerning the research, authorship, and/or publication of this article: Aku, Y.T., U.T., A.G., T.R., U.G., D.K., B.W., A.K., M.R., A.H.N. report no disclosures. M.E. reports grants from Bayer and Ipsen and fees for lectures and/or consulting paid to the Charité from Amgen, AstraZeneca, Bayer Healthcare, BMS, Daiichi Sankyo, all outside of the submitted work.

## Supporting information


**Figure S1:** Pearson correlation matrices of mobility and gait assessment variables (*N* = 94). (A) Pearson correlations among seven mobility‐related measures: EQ‐5D‐5L Mobility Subdomain, Rivermead Mobility Index (RMI), modified Rankin Scale (mRS), Barthel Index (BI)—Walking, BI—Stairs, BI—Transfers, and Walking Aid use (yes/no). (B) Pearson correlations among four gait‐specific measures: 10‐Meter Walk Test (10MWT) step count, 10MWT duration, 6‐Minute Walk Test (6MWT) distance, and gait speed. Color intensity reflects the magnitude and direction of the correlation coefficient, ranging from dark red (*r* = 1.00) to dark blue (*r* = −1.00). Correlation coefficients are displayed within each cell. All measures were assessed at baseline.
**Figure S2:** Principal component analysis (PCA) within each domain of mobility (above) and gait (below). For both domains, all variables load positively on PC 1, confirming that higher transformed scores reflect worse overall function. For mobility, PC 1 alone accounts for 56% of the total variance. For gait, PC1 alone accounts for 82% of the total variance. RMI: Rivermead Mobility Index; mRS: modified Rankin Scale; BI: Barthel Index; 10MWT: 10‐Meter Walk Test; 6MWT: 6‐Minute Walk Test.
**Figure S3:** Parallel analysis for component retention in both domains (mobility above and gait below). Observed eigenvalues (blue) are plotted against the distribution of eigenvalues from 5,000 randomly permuted datasets (orange dashed line with 5%–95% confidence band). Only the first principal component (PC1) exceeds the upper bound of the null distribution. The result confirms that PC1 captures meaningful variance beyond chance, justifying the use of PC1 alone in subsequent analyses.
**Table S1:** Transformation and Truncation Rules for Gait and Mobility Variables Used in PCA. Transformations were applied to ensure that higher values consistently indicated worse function, and scores were truncated at zero to prevent artificial negative values. These steps allowed for clinically interpretable, directionally aligned input variables for composite PC1 scores used in subsequent lesion‐network mapping.
**Table S2:** Patient demographics and clinical characteristics of the entire cohort and stratified based on primary outcome (fallers vs. non‐fallers).
**Table S3:** Clinical characteristics of fallers (*n* = 17). *Baseline* refers to the time of hospital admission. Treatment category: ***PT*** indicates physical training. ***R*** denotes relaxation. TOAST Classification: ***1***, large artery atherosclerosis; ***2***, cardioembolic stroke; ***3***, small vessel occlusion; ***4***, unknown etiology. Arterial territory abbreviations: ***VB***, vertebrobasilar arteries. Baseline EQ‐5D‐5L domains: ***A***, Anxiety/depression; ***M***, problems with mobility; ***P***, Pain/discomfort; ***D***, problems with activities of daily living; *S*, problems with self‐care. For each EQ‐5D‐5L domain, responses were dichotomized as indicating a problem if any level above 1 was endorsed (i.e., levels 2–5). ***VPA*** refers to verbal or physical assistance. NaN*: Did not attend the 6MWT.
**Table S4:** Univariable and multivariable binary logistic regression analysis for falls (*N* = 93; 17 fallers). Each multivariable model includes age, treatment group (intervention vs. control), and either the PC1‐Mobility or PC1‐Gait network spatial similarity score. Pseudo *R* ^2^ = McFadden's pseudo *R* ^2^; lower AIC indicates better model fit. Although higher age and intervention group assignment were associated with increased fall risk, individual lesion connectivity scores showed no significant association.
**Table S5:** The brain regions functionally connected to the uncorrected raw T‐score fall network.

## Data Availability

The data that support the findings of this study are available from the corresponding author upon reasonable request. Open source softwares were used for the pre‐processing and analysis of the data, including: Lead‐DBS (https://github.com/netstim/leaddbs), LESYMAP package in R version 4.2.0 (https://github.com/dorianps/LESYMAP), FSL version 6.0.6.4 (https://fsl.fmrib.ox.ac.uk/fsl/fslwiki/) and ANTsPy (https://github.com/ANTsX/ANTsPy).
