# Supplementary material for: Who Falls After a Stroke? Evidence From a Prospective Stroke Cohort
Source: Eur J Neurol. 2026 Jun 24;33(6):e70678. doi: 10.1111/ene.70678 (PMC13292268; doi:10.1111/ene.70678)
Supplement: Supplementary file 1 — Figure S1: Pearson correlation matrices of mobility and gait assessment variables (N = 94). (A) Pearson correlations among seven mobility‐related measures: EQ‐5D‐5L Mobility Subdomain, Rivermead Mobility Index (RMI), modified Rankin Scale (mRS), Barthel Index (BI)—Walking, BI—Stairs, BI—Transfers, and Walking Aid use (yes/no). (B) Pearson correlations among four gait‐specific measures: 10‐Meter Walk Test (10MWT) step count, 10MWT duration, 6‐Minute Walk Test (6MWT) distance, and gait speed. Color intensity reflects the magnitude and direction of the correlation coefficient, ranging from dark red (r = 1.00) to dark blue (r = −1.00). Correlation coefficients are displayed within each cell. All measures were assessed at baseline. Figure S2: Principal component analysis (PCA) within each domain of mobility (above) and gait (below). For both domains, all variables load positively on PC 1, confirming that higher transformed scores reflect worse overall function. For mobility, PC 1 alone accounts for 56% of the total variance. For gait, PC1 alone accounts for 82% of the total variance. RMI: Rivermead Mobility Index; mRS: modified Rankin Scale; BI: Barthel Index; 10MWT: 10‐Meter Walk Test; 6MWT: 6‐Minute Walk Test. Figure S3: Parallel analysis for component retention in both domains (mobility above and gait below). Observed eigenvalues (blue) are plotted against the distribution of eigenvalues from 5,000 randomly permuted datasets (orange dashed line with 5%–95% confidence band). Only the first principal component (PC1) exceeds the upper bound of the null distribution. The result confirms that PC1 captures meaningful variance beyond chance, justifying the use of PC1 alone in subsequent analyses. Table S1: Transformation and Truncation Rules for Gait and Mobility Variables Used in PCA. Transformations were applied to ensure that higher values consistently indicated worse function, and scores were truncated at zero to prevent artificial negative values. These steps allowed fo [file ENE-33-e70678-s001.docx]

- **SUPPLEMENTARY MATERIAL -**

**Who Falls After Stroke? Evidence From a Prospective Stroke Cohort**

Anna Kufner*, Yunyou Tang*, Uchralt Temuulen, Ghadir Abbas, Torsten Rackol, Ulrike Grittner, Daniel Kroneberg, Benedikt Weigel, Andrea A Kühn, Martin Reich, Alexander H Nave, Matthias Endres

| **Domain** | **Variable Name** | **Transformation Applied** | **Truncation Rule** |
| --- | --- | --- | --- |
| **Mobility** | EQ-5D-5L Mobility Subdomain | Used as is | None |
|  | RMI | 15 – observed value | None |
|  | mRS | Used as is | None |
|  | BI – Walking | 15 – observed value | Values ≤ 0 set to 0 |
|  | BI – Stairs | 10 – observed value | Values ≤ 0 set to 0 |
|  | BI – Transfers | 15 – observed value | Values ≤ 0 set to 0 |
|  | Walking Aid (yes = 1, no = 0) | Inverted → 1 = aid → 1, 0 = no aid → 0 | None |
| **Gait** | 10MWT – Steps | observed value – 12 | Values ≤ 0 set to 0 |
|  | 10MWT – Duration | observed value – 10 | Values ≤ 0 set to 0 |
|  | 6MWT – Distance | 300 – observed value | Values ≤ 0 set to 0 |
|  | Gait Speed (m/s) | 1.2 – observed value | Values ≤ 0 set to 0 |

**Supplementary Table S1:** Transformation and Truncation Rules for Gait and Mobility Variables Used in PCA. Transformations were applied to ensure that higher values consistently indicated worse function, and scores were truncated at zero to prevent artificial negative values. These steps allowed for clinically interpretable, directionally aligned input variables for composite PC1 scores used in subsequent lesion-network mapping.


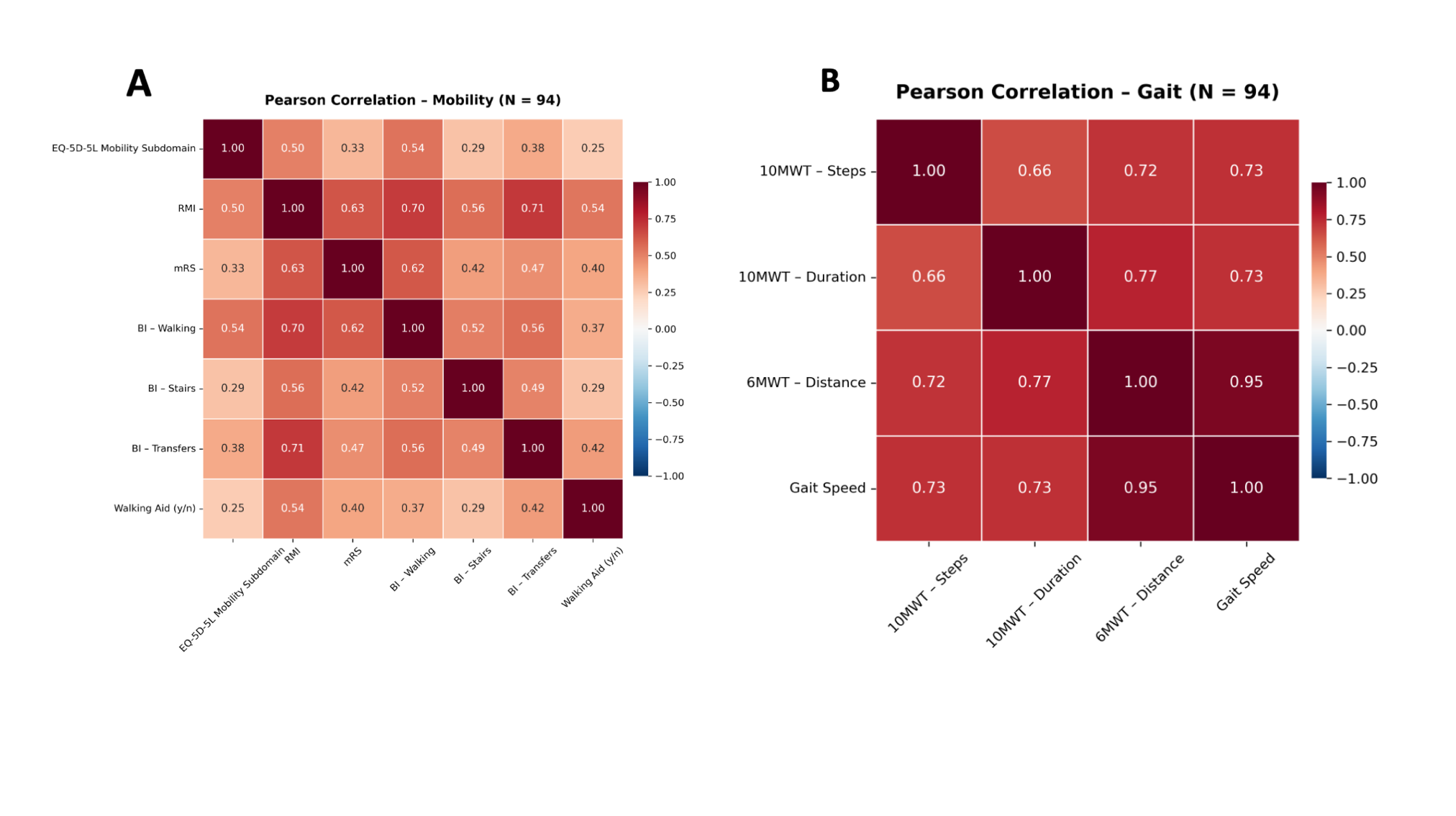


**Supplementary Figure S1:** **Pearson correlation matrices of mobility and gait assessment variables (N = 94). *A)*** Pearson correlations among seven mobility-related measures: EQ-5D-5L Mobility Subdomain, Rivermead Mobility Index (RMI), modified Rankin Scale (mRS), Barthel Index (BI) – Walking, BI – Stairs, BI – Transfers, and Walking Aid use (yes/no). ***B)*** Pearson correlations among four gait-specific measures: 10-Metre Walk Test (10MWT) step count, 10MWT duration, 6-Minute Walk Test (6MWT) distance, and gait speed. Color intensity reflects the magnitude and direction of the correlation coefficient, ranging from dark red (r = 1.00) to dark blue (r = −1.00). Correlation coefficients are displayed within each cell. All measures were assessed at baseline.


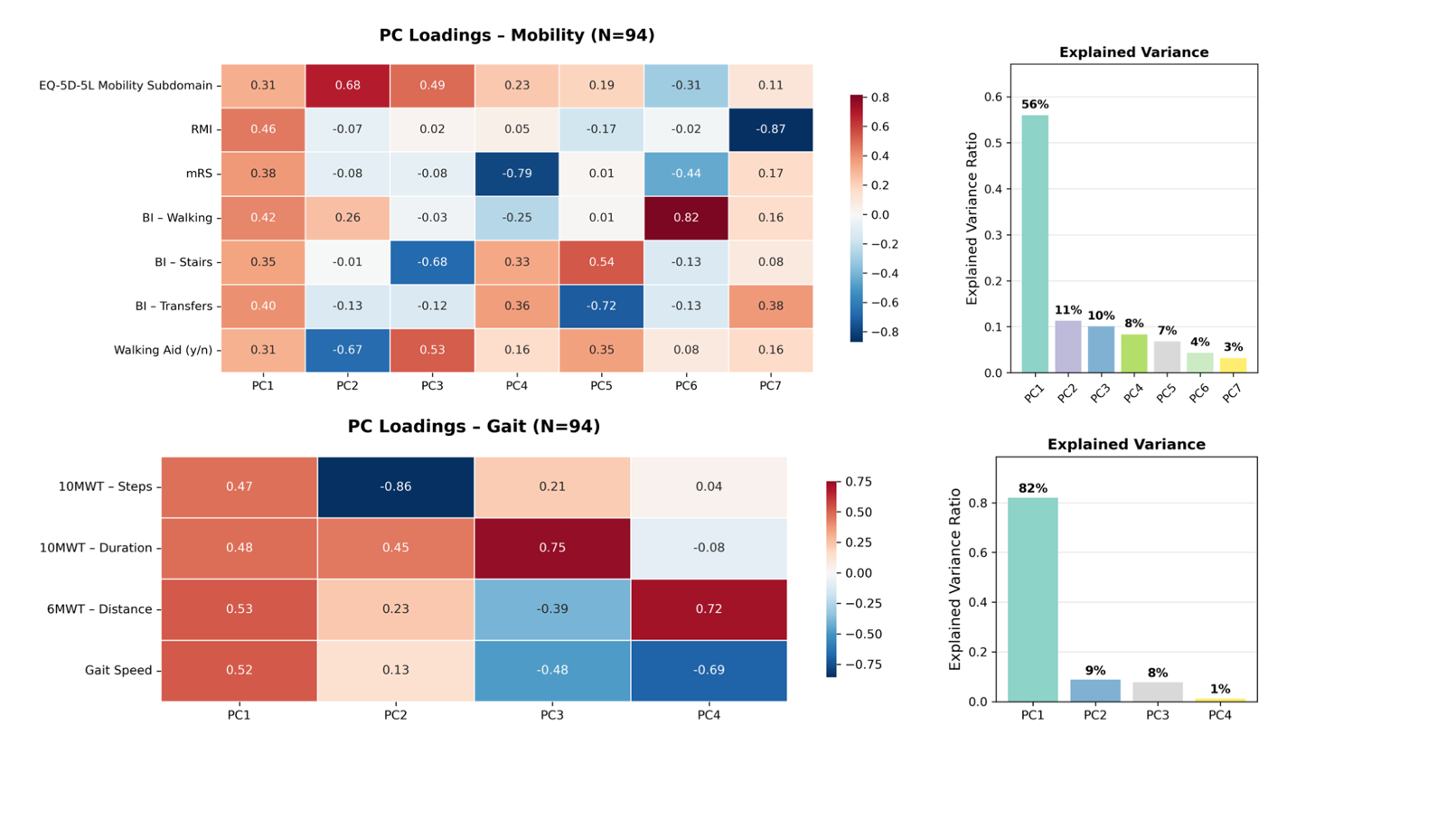


**Supplementary Figure S2:** Principal component analysis (PCA) within each domain of mobility (above) and gait (below). For both domains, all variables load positively on PC 1, confirming that higher transformed scores reflect worse overall function. For mobility, PC 1 alone accounts for 56 % of the total variance. For gait, PC1 alone accounts for 82% of the total variance. RMI: Rivermead Mobility Index; mRS: modified Rankin Scale; BI: Barthel Index; 10MWT: 10-Metre Walk Test; 6MWT: 6-Minute Walk Test.


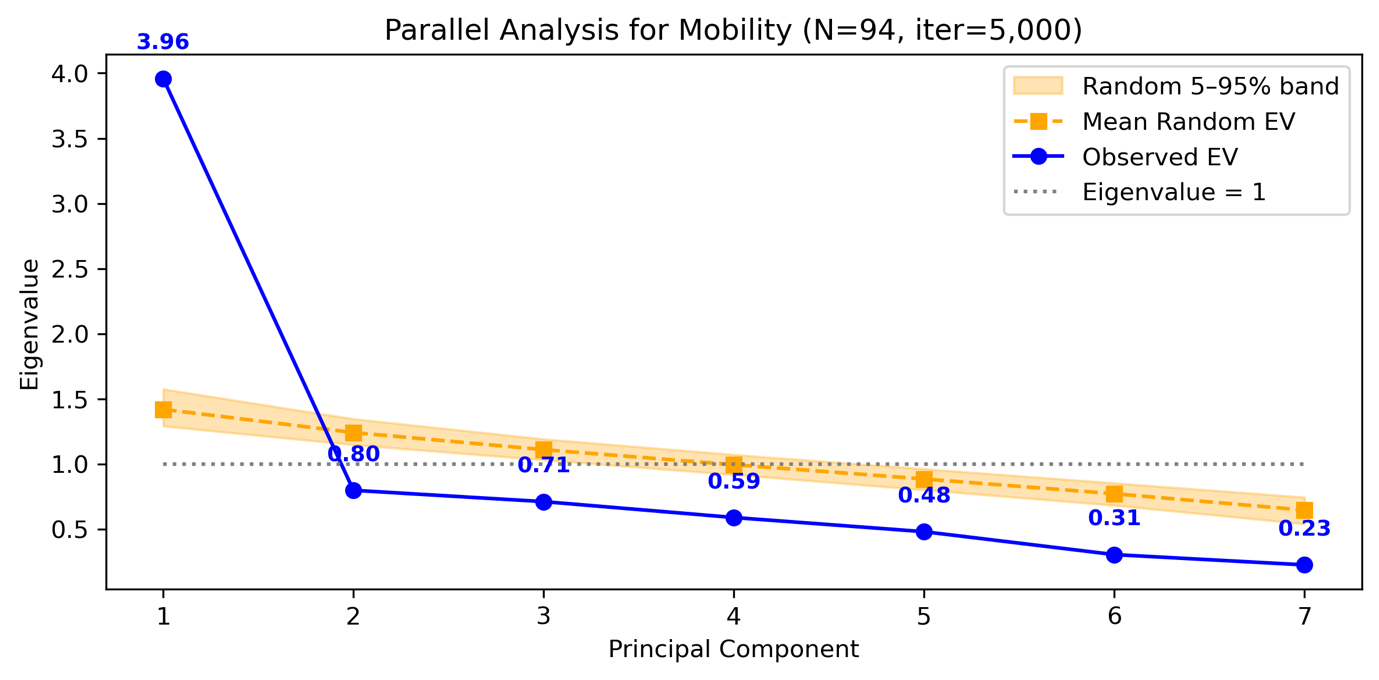


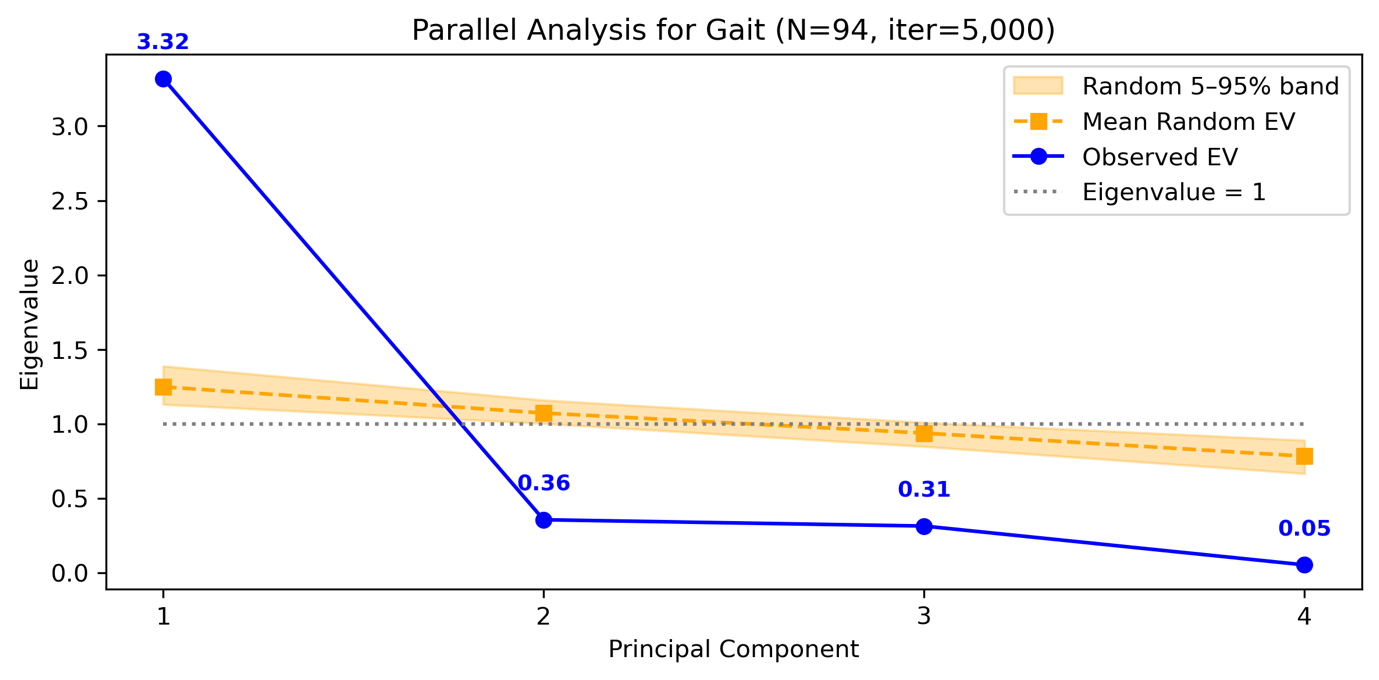


**Supplementary Figure S3:** Parallel analysis for component retention in both domains (mobility above and gait below). Observed eigenvalues (blue) are plotted against the distribution of eigenvalues from 5,000 randomly permuted datasets (orange dashed line with 5–95% confidence band). Only the first principal component (PC1) exceeds the upper bound of the null distribution. The result confirms that PC1 captures meaningful variance beyond chance, justifying the use of PC1 alone in subsequent analyses.

|  | **BAPTISe** | **Faller** | **Non_faller** | **P values** |
| --- | --- | --- | --- | --- |
|  | **N=94** | **N=17** | **N=76** |  |
| Age, mean (±SD) | 68.7 (11.0) | 74.8 (8.7) | 67.1(10.9) | 0.0069** |
| Sex, female, n (%) | 42 (44.7) | 7 (41.2) | 34 (44.7) | 0.516 |
| Hypertension, n (%) | 80 (85.1) | 16 (94.1) | 63 (82.9) | 0.459 |
| Diabetes, n (%) | 26 (27.7) | 5 (29.4) | 21 (27.6) | 0.815 |
| Hyperlipidemia, n (%) | 46 (48.9) | 8 (47.1) | 37 (48.7) | 0.586 |
| Atrial fibrillation, n (%) | 16 (17.0) | 4 (23.5) | 11 (14.5) | 0.057 |
| History of smoking, n (%) | 35 (37.2) | 5 (29.4) | 29 (38.2) | 0.34 |
| History of stroke, n (%) | 14 (14.9) | 4 (23.5) | 10 (13.2) | 0.508 |
| Post-stroke time window, median (IQR) | 25.5 (14-33) | 17 (11-33) | 27 (15.5-33) | 0.31 |
| REPAS spasticity score at 3 months, median (IQR) | 6 (2-13) | 12 (2-14) | 6 (2-11) | 0.396 |
| Treatment, n (%) | 53 (56.4) | 13 (76.5) | 39 (51.3) | 0.113 |
| TOAST Classification (Adams 1993), 2 missing |  |  |  | 0.496 |
| Large artery atherosclerosis, n (%) | 16 (17.4) | 3 (17.7) | 13 (17.6) |  |
| Cardioembolic stroke, n (%) | 14 (15.2) | 4 (23.5) | 9 (12.2) |  |
| Small vessel occlusion, n (%) | 21 (22.8) | 6 (35.3) | 15 (20.3) |  |
| Other, n (%) | 3 (3.3) | 0 | 3 (4.1) |  |
| Unknown, n (% | 36 (38.1) | 4 (23.5) | 32 (43.2) |  |
| ARWMC score, median (IQR) | 6 (4-10) | 8 (6-14) | 5 (4-8) | 0.0084* |
| Lesion volume at baseline (ml), median (IQR) | 11.04 (2.9-54.3) | 8.9 (3.8-53.5) | 14.6 (3.0-54.3) | 0.8781 |
| NIHSS at admission, median (IQR) | 9 (6-12) | 7 (4-12) | 9 (6-12) | 0.913 |
| Arterial territory (Refrence the atlas) |  |  |  | 0.979 |
| Middle Cerebral Artery (MCA), n (%) | 0 | 0 | 0 |  |
| Anterior Cerebral Artery (ACA), n (%) | 0 | 0 | 0 |  |
| Posterior Cerebral Artery (PCA), n (%) | 1 (1.1) | 0 | 1 (1.3) |  |
| Vertebrobasilar arteries (VB), n (%) | 9 (9.6) | 2 (11.8) | 7 (9.2) |  |
| Multiple arterial territories, n (%) | 84 (89.4) | 15 (88.2) | 68 (89.5) |  |
| Baseline EQ-5D-5L |  |  |  |  |
| Anxiety, n (%) | 42 (44.7) | 7 (41.2) | 35 (46.1) | 0.676 |
| Mobility, n (%) | 85 (90.4) | 15 (88.2) | 70 (92.1) | 0.007* |
| Pain, n (%) | 56 (59.6) | 7 (41.2) | 49 (64.5) | 0.237 |
| Problems in daily life activities, n (%) | 85 (90.4) | 14 (82.4) | 70 (92.1) | 0.714 |
| Problems in self care, n (%) | 78 (83.0) | 13 (76.5) | 64 (84.2) | 0.779 |

**Supplementary Table S2:** Patient demograpahics and clinical characteristics of entire cohort and stratified based on primary outcome (fallers versus non-fallers).

| **Fallers** | **Age** | **Sex** | **REPAS spasticity score at 3 months** | **Treatment** | **ARWMC score** *(Baseline)* | **Lesion volume at baseline (ml)** | **NIHSS at admission** | **Baseline EQ-5D-5L** *(Baseline)* | **mRS***(Base-line)* | **BI – Walking**  *(Baseline)* | **BI – Transfers**  *(Baseline)* | **Walking Aid during 6MWT** *(Baseline)* |
| --- | --- | --- | --- | --- | --- | --- | --- | --- | --- | --- | --- | --- |
| **1** | 85 | F | NaN | *R* | 4 | 0.56 | 9 | *M, P, D, S* | 3 | *VPA* | Minor *VPA* | Yes |
| **2** | 74 | M | 28 | *PT* | 8 | 2.911 | 5 | *M, D, S* | 4 | Wheelchair | Major *VPA* | NaN* |
| **3** | 79 | F | NaN | *PT* | 8 | 9.188 | 4 | *M, D, S* | 3 | *VPA* | Minor *VPA* | Yes |
| **4** | 67 | M | 2 | *PT* | 4 | 13.32 | 4 | *M, S* | 3 | Wheelchair | Minor *VPA* | Yes |
| **5** | 85 | F | 4 | *PT* | 15 | 11.669 | 10 | *A, M, P, D, S* | 4 | *VPA* | Minor *VPA* | Yes |
| **6** | 74 | M | 14 | *R* | 6 | 8.578 | 6 | *M, P, D, S* | 3 | *VPA* | Minor *VPA* | Yes |
| **7** | 77 | M | 19 | *PT* | 20 | NaN | 4 | *M, D, S* | 4 | Not Mobile | Minor *VPA* | Yes |
| **8** | 83 | F | 0 | *PT* | 11 | 1.141 | 7 | *A, M, D, S* | 4 | Wheelchair | Minor *VPA* | Yes |
| **9** | 85 | F | 12 | *PT* | 8 | 8.022 | 4 | *A, M, P, D, S* | 4 | Not Mobile | Major *VPA* | Yes |
| **10** | 60 | M | 0 | *R* | 0 | 4.738 | 6 | *M, P, D, S* | 3 | Wheelchair | Independent | Yes |
| **11** | 80 | F | NaN | *PT* | 18 | 143.662 | 12 | *A, M, P, D, S* | 4 | Not Mobile | Major *VPA* | Yes |
| **12** | 55 | M | 13 | *PT* | 8 | 6.852 | 12 | *A, M, D, S* | 4 | Not Mobile | Major *VPA* | Yes |
| **13** | 75 | F | 14 | *PT* | 22 | 45.297 | 14 | *A, M, D, S* | 4 | *VPA* | Minor *VPA* | Yes |
| **14** | 70 | M | 26 | *PT* | 13 | 61.636 | 12 | *NaN* | 4 | Not Mobile | Major *VPA* | Yes |
| **15** | 74 | M | 7 | *R* | 4 | 73.506 | 8 | *A, D, S* | 4 | *VPA* | Major *VPA* | Yes |
| **16** | 80 | M | 0 | *PT* | 14 | 1.945 | 4 | *M, D, S* | 4 | Wheelchair | Major *VPA* | Yes |
| **17** | 68 | M | NaN | *PT* | 12 | 203.994 | 14 | *M, P, S* | 4 | Not Mobile | Major *VPA* | NaN* |

**Supplementary Table S3.** Clinical characteristics of fallers (n = 17). *Baseline* refers to the time of hospital admission. Treatment category: ***PT*** indicates physical training. ***R*** denotes relaxation. TOAST Classification: ***1****,* large artery atherosclerosis; ***2****,* cardioembolic stroke; ***3****,* small vessel occlusion; ***4****,* unknown etiology. Arterial territory abbreviations: ***VB***, vertebrobasilar arteries. Baseline EQ-5D-5L domains: ***A***, Anxiety/depression; ***M***, problems with mobility; ***P***, Pain/discomfort; ***D***, problems with activities of daily living; ***S****,* problems with self-care. For each EQ-5D-5L domain, responses were dichotomized as indicating a problem if any level above 1 was endorsed (i.e., levels 2–5). ***VPA*** refers to verbal or physical assistance. NaN*: Did not attend the 6MWT.

|  | **Univariable** | | **Multivariable** | |
| --- | --- | --- | --- | --- |
|  | **Odds ratio** | **p-value** | **Odds ratio** | **p-value** |
| **Age (*cont*)** | 1.08(1.02-1.15) | 0.013* | 1.08 (1.02-1.16) | 0.01* |
| **Treatment group (*cat*)** | 3.08 (0.92-10.31) | 0.068* | 3.98 (1.09-14.57) | 0.037* |
| **PC1-Mobility spatial similarity score (*cont*)** | 1.23 (0.31-4.87) | 0.771 | 1.74 (0.39-7.75) | 0.466 |

*For multivariable model, Pseudo R^2^ = 0.145, AIC = 83.67.*

|  | **Univariable** | | **Multivariable** | |
| --- | --- | --- | --- | --- |
|  | **Odds ratio** | **p-value** | **Odds ratio** | **p-value** |
| **Age (*cont*)** | 1.08(1.02-1.15) | 0.013* | 1.08 (1.02-1.16) | 0.01* |
| **Treatment group (*cat*)** | 3.08 (0.92-10.31) | 0.068* | 3.67 (1.03-13.04) | 0.044* |
| **PC1-Gait spatial similarity score (*cont*)** | 0.85 (0.19-3.83) | 0.834 | 0.79 (0.163.98) | 0.78 |

*For multivariable model, Pseudo R2 = 0.139; AIC = 84.14.*

**Supplementary Table S4.** Univariable and multivariable binary logistic regression analysis for falls (N = 93; 17 fallers). Each multivariable model includes age, treatment group (intervention vs. control), and either the PC1-Mobility or PC1-Gait network spatial similarity score. Pseudo R² = McFadden’s pseudo R²; lower AIC indicates better model fit. Although higher age and intervention group assignment were associated with increased fall risk, individual lesion connectivity scores showed no significant association.

|  | **HOOC** | **HOOS** | **Atlas of the Human Cerebellum** |
| --- | --- | --- | --- |
| **Fall network** | Insular Cortex | Right Thalamus | Left VI |
|  | Precentral Gyrus |  |  |
|  | Postcentral Gyrus |  |  |
|  | Superior Parietal Lobule |  |  |
|  | Supramarginal Gyrus, anterior division |  |  |
|  | Supramarginal Gyrus, posterior division |  |  |
|  | Lateral Occipital Cortex, superior division |  |  |
|  | Lateral Occipital Cortex, inferior division |  |  |
|  | Juxtapositional Lobule Cortex (formerly Supplementary Motor Cortex) |  |  |
|  | Precuneous Cortex |  |  |
|  | Central Opercular Cortex |  |  |
|  | Parietal Operculum Cortex |  |  |

**Supplementary Table S5.** The brain regions functionally connected to uncorrected raw T-score fall network.
